# Disentangling contributions to guest binding inside a coordination cage host: analysis of a set of isomeric guests with differing polarities†

**DOI:** 10.1039/d2dt02623f

**Published:** 2022-09-16

**Authors:** Cristina Mozaceanu, Atena B. Solea, Christopher G. P. Taylor, Burin Sudittapong, Michael D. Ward

**Affiliations:** Department of Chemistry, University of Warwick Coventry CV4 7AL UK m.d.ward@warwick.ac.uk

## Abstract

Binding of a set of three isomeric guests (1,2-, 1,3- and 1,4-dicyanobenzene, abbreviated DCB) inside an octanuclear cubic coordination cage host **H** (bearing different external substitutents according to solvent used) has been studied in water/dmso (98 : 2) and CD_2_Cl_2_. These guests have essentially identical molecular surfaces, volumes and external functional groups to interact with the cage interior surface; but they differ in polarity with dipole moments of *ca.* 7, 4 and 0 Debye respectively. In CD_2_Cl_2_ guest binding is weak but we observe a clear correlation of binding free energy with guest polarity, with 1,4-DCB showing no detectable binding by NMR spectroscopy but 1,2-DCB having −Δ*G* = 9 kJ mol^−1^. In water (containing 2% dmso to solubilise the guests) we see the same trend but all binding free energies are much higher due to an additional hydrophobic contribution to binding, with −Δ*G* varying from 16 kJ mol^−1^ for 1,4-DCB to 22 kJ mol^−1^ for 1,4-DCB: again we see an increase associated with guest polarity but the increase in −Δ*G* per Debye of dipole moment is around half what we observe in CD_2_Cl_2_ which we ascribe to the fact the more polar guests will be better solvated in the aqueous solvent. A van't Hoff analysis by variable-temperature NMR showed that the improvement in guest binding in water/dmso is entropy-driven, which suggests that the key factor is not direct electrostatic interactions between a polar guest and the cage surface, but the variation in guest desolvation across the series, with the more polar (and hence more highly solvated) guests having a greater favourable entropy change on desolvation.

## Abstract 2

The three dicyanobenzene isomers have obvious similarities but differ in their dipole moment: effects on binding in a coordination cage host in different solvents are discussed.

## Graphical abstract

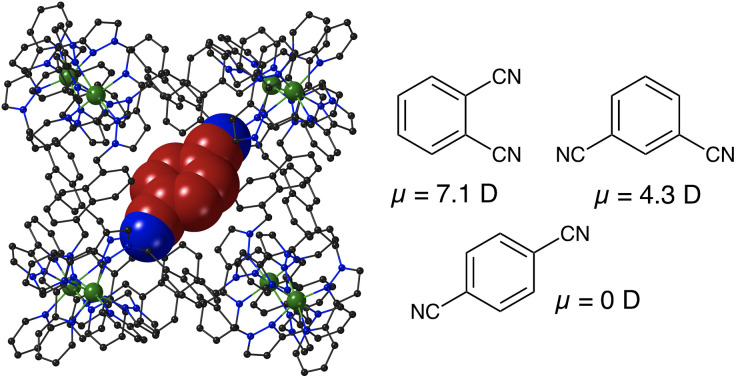

## Introduction

The ability of coordination cages^1^ to bind guest molecules in their cavities underpins a range of useful applications.^2–5^ The most widespread of these is catalysis, with the unusual environment inside the cavity providing a basis for altered reactivity of bound guests with – in some cases – spectacular rate accelerations of reactions that are comparable to what can be demonstrated by enzymes that have been evolved for that very specific purpose.^2–4^ Also important are applications relating to reversible uptake and release associated with transport of molecular ‘cargoes’^5^ and multi-step cascade processes.^6^ The size and shape specificity of coordination cages hosts based on their cavity dimensions also lends itself to them being used analytically as sensors to detect the presence of specific guests.^7^

The factors responsible for binding of guests to hosts in supramolecular assemblies have been extensively reviewed.^8^ However it is fair to say that in the specific subset of host/guest chemistry associated with metal complex coordination cages, detailed analyses of factors responsible for guest binding are relatively limited: usually guests are evaluated for binding and, if they bind, association constants can be determined as a prelude to studying the desired practical applications. Knowing binding constants is of course important but this information alone falls short of providing detailed insight into the factors that are responsible for guest binding in the way that has long been routinely applied to understanding biological or organic host/guest systems.^8^ An early quantitative analysis was based on relative sizes of host and guest by Rebek and became the basis of the so-called ‘55% rule’.^9^ Beyond that, systematic analyses of thermodynamic factors responsible for guest binding in cage hosts are rather limited, with the work of Raymond and co-workers providing the most prominent examples.^10^

**Fig. 1:**
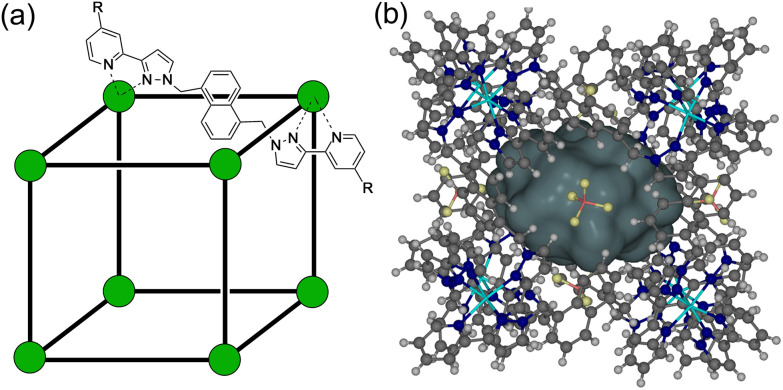
(a) A sketch of the cubic host cage [M_8_L_12_]^16+^, abbreviated as **H** (R = H), emphasising the cubic array of Co(ii) ions and the disposition of one bridging ligand; and its derivatives bearing substituents at the twenty-four externally-directed pyridyl C^4^ positions **H**^**W**^ (R = CH_2_OH), and **H**^**PEG**^ [R = –(CH_2_OCH_2_)_3_CH_2_OMe]. (b) A view of the complete cage structure, highlighting the cavity space (*V* = 409 Å^3^) where guests can bind.

In our own work, which has focussed in particular on analysis of guest binding inside an octanuclear cubic [M_8_L_12_]^16+^ host **H** (Fig. 1),^4*a*^ we have delved into the specific thermodynamic factors responsible for guest binding in different solvents in some detail.^11–15^ This has involved varying one parameter across a guest series at a time and examining the effects. Thus, evaluation of binding of a series of guests of the same shape and size but with different H-bond acceptor capabilities allowed us to quantify the contribution of hydrogen-bonding between guest and the cage interior surface as a contribution to guest binding.^11^ Evaluation of guest binding in water of matched pairs of guests with or without an additional fused aromatic ring,^12^ and with or without an additional CH_2_ group,^13,14^ allowed us to determine the hydrophobic contribution to guest binding as a function of hydrophobic surface area associated with aryl and alkyl substituents. Temperature-dependent measurements of guest binding highlighted the enthalpy and entropy contributions to the hydrophobic effect associated with liberation of water molecules from a confined pseudo-spherical cavity.^14^ Comparison of guests containing branched *vs.* linear alkyl chains, which differ in their number of freely rotatable bonds, highlighted entropy effects associated with loss of conformational flexibility on binding.^15^ The result of all this has been development of an empirical predictive model for guest binding that allows identification of new guests and prediction of their binding strength in water with a high degree of confidence: this predictive tool, based on the protein/ligand docking software ‘GOLD’ but with a customised scoring function based on the coordination cage **H** as ‘host’ instead of a protein, has been invaluable in our subsequent work on cage-based host/guest chemistry.^16,17^

To pursue our understanding of guest binding further we were interested to examine the effects of guest polarity in different solvents, as manifested in the guest dipole moment. This is quite different from the effects of specific (charge-assisted) hydrogen-bonding interactions between cage and guest which are highly dependent on the local functional groups involved in the interaction.^11^ To study polarity effects we have examined guest binding properties of a series of isomeric guests, specifically the three isomers of dicyanobenzene (DCB). All three have essentially the same molecular volume and are comfortably small enough to bind inside the cage cavity, as crystal structures show. They all have essentially the same surface area, meaning that the surface matching with the interior of the cage will liberate the same number of water molecules from both surfaces, leading to similar hydrophobic contributions to binding. And all have two nitrile functional groups which are weak hydrogen-bond acceptors. However, the differences in polarity – as expressed in their dipole moments – are significant with calculated gas-phase dipole moments of 7.1 (1,2-isomer), 4.3 (1,3-isomer) and 0 Debye (1,4 isomer).^18^ Comparison of the three guests should therefore reveal the extent to which a molecular dipole influences guest binding in the cage different solvents – water and CH_2_Cl_2_ – as this variable is the main difference across this guest series. Overall the work we describe here contributes to our understanding of optimising guest binding in synthetic hosts with a view to increased predictability.

## Results and discussion

### Structures of the cages used

The unsubstituted cage **H** that we reported originally,^19^ which was soluble only in polar organic solvents, can be functionalised with differing external substituents to control solubility. For this paper we have used **H** (devoid of external substituents) for the crystallographic studies. The cage **H**^**w**^ incorporates hydroxymethyl substituents at the C4 position of every pyridine ring, conferring improved water solubility due to the set of 24 externally-directed OH groups.^12^ The cage **H**^**PEG**^ similarly has a set of 24 tri-ethyleneglycol monomethyl ether substituents, which confer much improved solubility compared to **H** in both water and organic solvents such as CH_2_Cl_2_ because of the flexibility of the PEG chains which allows them to adopt both polar or non-polar conformations as required.^20^ The structures of these are summarised in Fig. 1. Importantly the basic core structure (and cavity properties for guest binding purposes) are comparable in all cases, it is only external substituents that change.

### Crystal structures of cage/guest complexes

The crystal structures of the cage/guest complexes with each of the three guests were obtained using the crystalline sponge method,^21,22^ recently popularised by Fujita and co-workers,^21^ that we have employed recently with considerable success.^23^ Growing crystals of the cage **H** from solvent in the presence of guests generally leads to crystals of empty **H**; however, pre-preparing good-quality crystals of **H** solvothermally, and then immersing them in a concentrated solution of the desired guest (or the pure guest if it is a liquid) for a few hours, affords a good chance of the guest being taken up into the cage crystals without loss of crystallinity. Using this method we were able to perform single-crystal structural determinations of all three cage/DCB complexes which we denote **H**·**12DCB**, **H**·**13DCB** and **H**·**14DCB** with 1,2-, 1,3- and 1,4-DCB respectively as the guests.

**Fig. 2:**
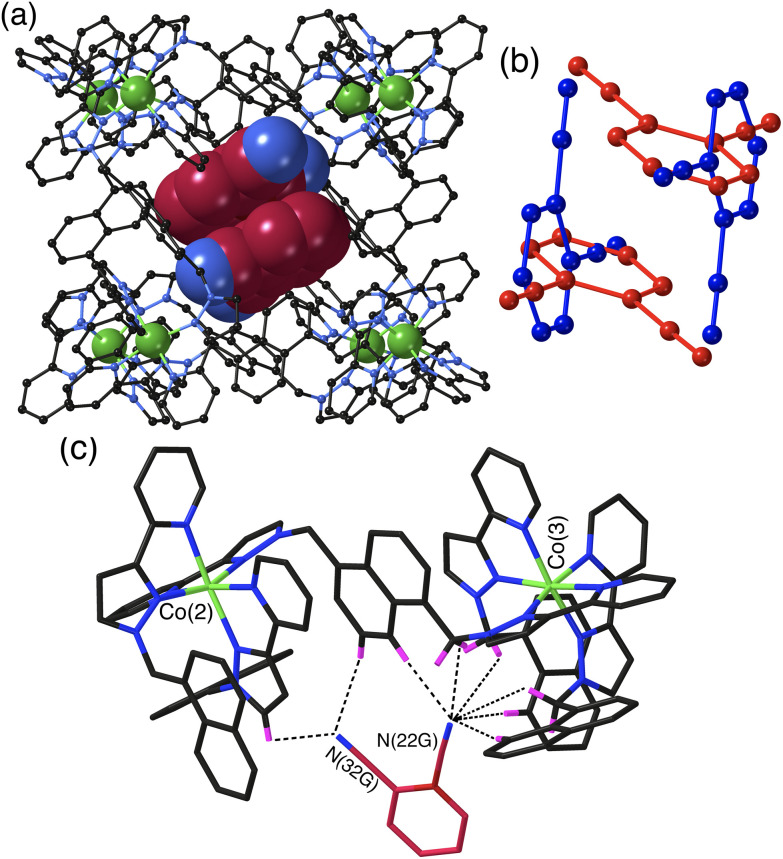
Crystal structure of **H**·**1,2DCB**. (a) View of the host with a centrosymmetric stacked pair of crystallographically equivalent 1,2-DCB guests shown in space-filling mode – this is the major disorder component with site occupancy of 0.88 in each of the two sites. (b) Illustration of the guest arrangement with major (red) and minor (blue) components, both forming centrosymmetric stacked pairs. (c) A view of the immediate hydrogen-bonding environment around one of the 1,2-DCB guests (major disorder component): nearby CH protons from the ligand array are highlighted in purple, with CH⋯N contacts to the nitrile groups of the guest of <3 Å shown by black dashed lines.

The crystal structure of **H**·**12DCB** (Fig. 2) reveals a stacked pair of 1,2-DCB guests in the cavity, lying astride an inversion centre such that they are crystallographically equivalent and their local dipoles cancel: Fujita and co-workers reported that in a stacked set of three aromatic guests inside a cage host the individual molecules in the stack were successively rotated by 120° with respect to their neighbours such that the individual molecular dipoles exactly cancelled.^24^ The stacked pair of 1,2-DCB guests is disordered over two orientations, with major and minor components having site occupancies of 0.88 and 0.12 (Fig. 2b): in the major pair the stacking distance between parallel aromatic rings is 3.42 Å, in the minor pair it is 3.46 Å. We have seen stacked pairs of aromatic guests before in many instances.^23,25^ In this case the presence of two guests is facilitated by the relatively small size of 1,2-DCB, such that the presence of two (combined volume 276 Å^3^) gives a cavity occupancy of 67%. As usual in cases where the guest has one or more externally-directed lone pairs, weak hydrogen-bonding interactions with the cage interior surface serve to orient the guest in the cavity (Fig. 2c). In particular one of the N atoms (N22G) is directed into a pocket close to a *fac* tris-chelate metal centre [Co(3)] where several C–H hydrogen atoms converge: as these are close to a Co(ii) centre in a region of positive electrostatic potential they carry a higher δ+ than a neutral CH group and participate in CH⋯N interactions,^11^ and N22G makes contacts of <3 Å with six CH protons from the methylene (CH_2_) or naphthyl protons in the surrounding pocket. The other N atom (N32G) makes a smaller number of contacts, with two significant CH⋯N interactions of 2.70 and 2.82 Å with naphthyl and pyrazolyl CH groups, respectively, close to the adjacent metal centre Co(2) (Fig. 2c). The low site occupancy of the minor disorder component [12%, see Fig. 2(b)] means that detailed analysis of its cage/guest contacts is unjustified.

**Fig. 3:**
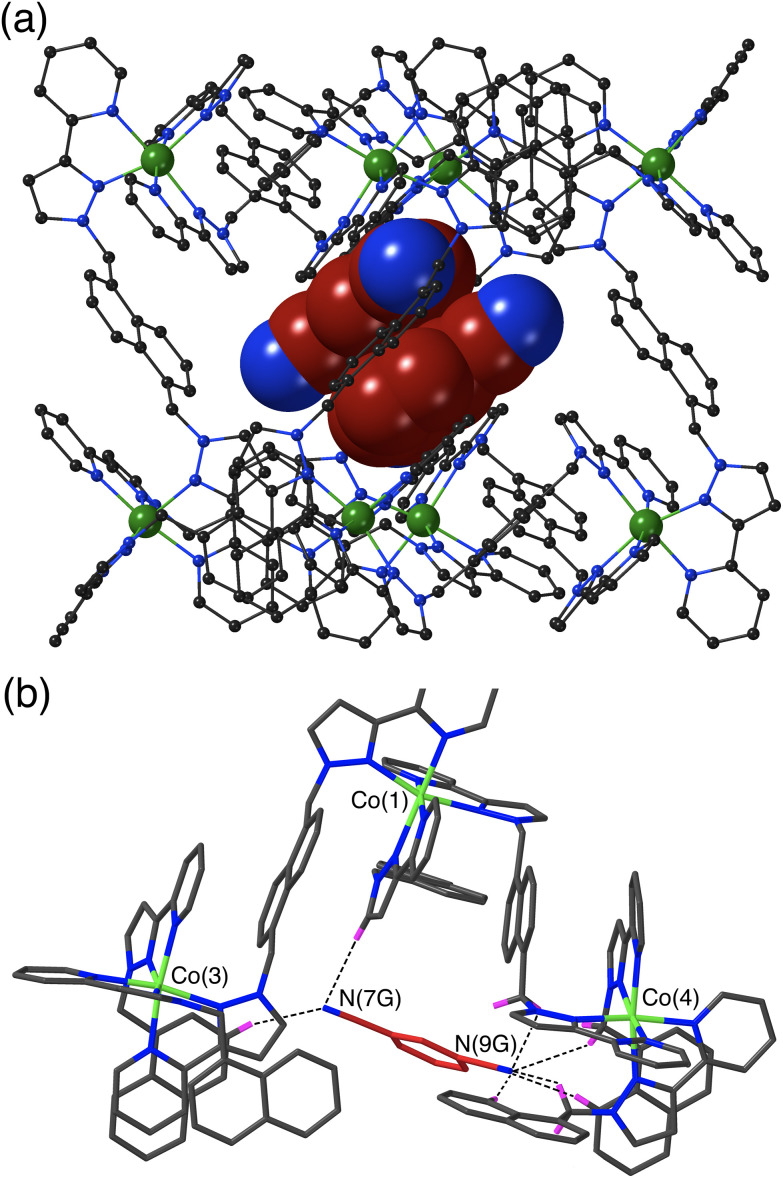
Crystal structure of **H**·**1,3DCB**. (a) View of the complete host with a centrosymmetric stacked pair of crystallographically equivalent 1,3-DCB guests shown in space-filling mode. (b) A view of the immediate hydrogen-bonding environment around one of the 1,3-DCB guests: nearby CH protons from the ligand array are highlighted in purple, with CH⋯N contacts to the nitrile groups of the guest of <3 Å shown by black dashed lines.

The crystal structure of **H**·**13DCB** likewise contains a stacked pair of guests, with unit site occupancy each and no positional disorder, lying astride an inversion centre such that their local dipoles cancel (Fig. 3a).^24^ The separation between the mean planes of the aromatic rings is 3.39 Å. Again, one of the weakly Lewis basic N atoms of the guest (N9G) projects into the convergent pocket of CH hydrogen atoms close to the *fac* tris-chelate metal centre Co(4), with four CH⋯N contacts in the range 2.51–2.84 Å. The other nitrile N atom N7G forms CH⋯N interactions of 2.52 and 2.84 Å with naphthyl and pyrazolyl CH protons in the vicinity of the adjacent metal ion Co(3) (Fig. 3b).

**Fig. 4:**
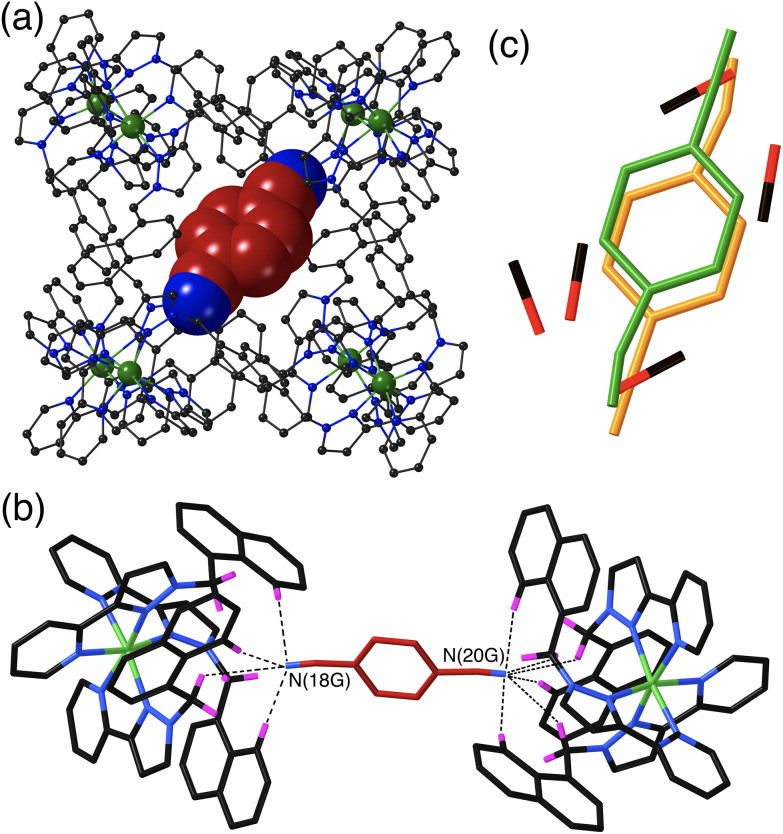
Crystal structure of **H**·**1,4DCB**. (a) View of the complete host with a single 1,4-DCB guest shown in space-filling mode. (b) A view of the immediate hydrogen-bonding environment around one of the 1,4-DCB guests: nearby CH protons from the ligand array are highlighted in purple, with CH⋯N contacts to the nitrile groups of the guest of <3 Å shown by black dashed lines. (c) Illustration of the disorder, with the cavity containing either one guest molecule disordered over two closely spaced but equivalent sites (total occupancy 0.5), or a set of six MeOH molecules (total occupancy 0.5). This disorder means that the 1,4-DCB guest is not centred within the cavity but lies slightly closer to one Co(ii) ion than the other along the cube diagonal in the view in part (b).

The crystal structure of **H**·**14DCB** (Fig. 4) is fundamentally different from the other two in that it contains a single 1,4-DCB guest in the cage cavity, disordered over two closely spaced positions either side of the inversion centre with a site occupancy of 0.25 in each, such that the overall occupancy of the cavity by 1,4-DCB is 50%. In addition there are six MeOH molecules, three in each asymmetric unit with site occupancies of 0.4, 0.45 and 0.65, hence three MeOH molecules in total in the cavity. The positions of the MeOH molecules substantially overlap with the position of the 1,4-DCB guest giving a range of unphysical inter-atomic distances (Fig. 4c), implying that the cavity contains *either* a 1,4-DCB guest (50% of the time) *or* six MeOH molecules (the other 50% of the time). Short O⋯O contacts [O(11S) – O(13S), 2.53 Å; and O(13S) – O(15S), 2.69 Å] are indicative of the presence of OH⋯O hydrogen bonds between the methanol molecules. The most significant feature of the structure is the orientation of the 1,4-DCB guest which lies along a long diagonal of the interior cavity (Fig. 4a) such that each nitrile N atom lies in the H-bond donor pocket associated with one of the *fac* tris-chelate sites [Co(4) and its symmetry equivalent; Fig. 4b]. The guest is not exactly centred in the cavity, but lies closer to one Co(4) than the other, with N(20G)⋯Co(4) and N(18G)⋯Co(4) separations of 5.16 and 5.66 Å respectively (of course the other disorder component is offset in the opposite sense giving an overall crystallographically centrosymmetric assembly). The length of 1,4-DCB (7.4 Å between the terminal N atoms) is close to optimal for spanning the cage long diagonal in this way, and indeed we observed a similar structure with the guest 1,2,4,5-tetracyanobenzene which was being studied as a guest for its powerful electron-accepting properties and their effect on cage photophysics.^26^ The resulting array of CH⋯N contacts between the guest and the cage interior surface (Fig. 4b) involves the methylene (CH_2_) and naphthyl protons in the binding pockets. Guest atom N(20G), which lies further into the pocket and closer to Co(4), has five CH⋯N contacts in the range 2.47–2.54 Å; guest atom N(18G), which lies slightly further out of the other pocket, has two comparably short interactions (2.46 and 2.55 Å) and a larger number of slightly longer ones.

The set of three structures has some obvious similarities in respect of the CH⋯N contacts between guest and cage interior surface. The relatively compact shapes of guests 1,2-DCB and 1,3-DCB allows a π-stacked pair to occupy the cage cavity giving a cavity occupancy in the solid-state of *ca.* 67%. In contrast the more elongated shape of 1,4-DCB seems to preclude this, with 1,4-DCB needing to lie along a long diagonal of the cavity to fit – an orientation which results in hydrogen-bonding interactions at both ends of the guest but which prevents the presence of a stacked pair. The cavity is therefore less efficiently filled by 1,4-DCB which may explain why only half of the cages contain a 1,4-DCB guest, with the other half containing a hydrogen-bonded network of MeOH molecules.

### Guest binding in solution: contributions to binding in different solvent systems

We next measured the binding constants of three guests in water using the hydroxymethyl-substituted cage **H**^**w**^ as the water-soluble host.^12^ To get an initial idea of binding constants – to allow us to determine whether we should be using NMR or UV/vis spectroscopy, for example – we first calculated binding constants using the molecular docking programme GOLD with our customised scoring function that was obtained from a large training set of empirical data.^16^ This afforded estimated log *K* values for 1 : 1 host/guest complex formation of 3.2, 3.1 and 1.8 for the 1,2-, 1,3- and 1,4-DCB isomers respectively, with a significantly lower binding constant predicted for 1,4-DCB compared to the other two. Binding constants in the region 10^2^–10^3^ M^−1^ ideally require mM concentrations of host and guest to get a high degree of binding, which means that NMR spectroscopy was selected as the most appropriate analytical technique.

Initial tests showed that the DCB isomers are not sufficiently soluble in water for this to be possible, however inclusion of a small amount of dmso (98% water, 2% dmso) cured the problem. We have avoided as far as possible using mixed solvent systems because selective solvation effects can have consequences for supramolecular interactions which are highly non-linear with solvent composition, as Hunter and co-workers have thoroughly demonstrated.^27^

However use of just 2% dmso in water fixed the solubility problems and still gave binding constants of the same order of magnitude (and in the same relative ordering) as those predicted using GOLD (see below).

**Fig. 5:**
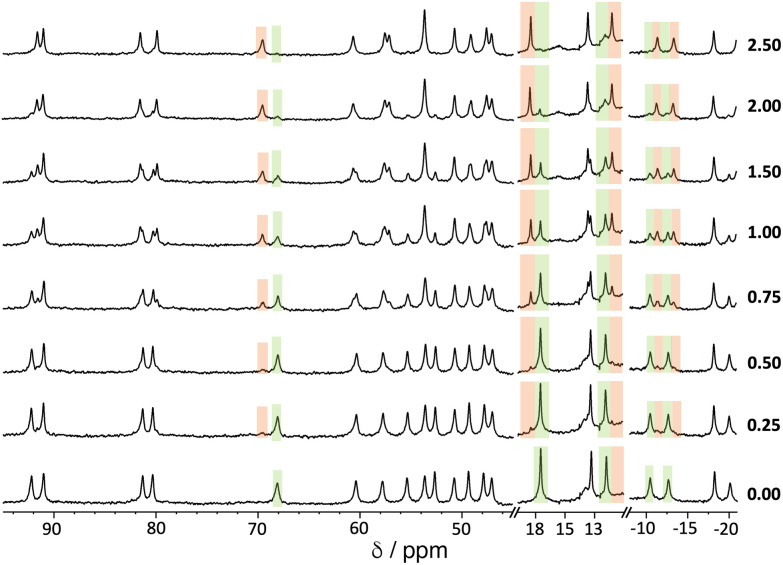
^1^H NMR titration experiment showing evolution of the NMR spectra of **H**^**w**^ (0.25 mM, 298 K) in D_2_O/DMSO-d^6^ (98 : 2, *v*/*v*) on addition of increasing amounts of 1,2-DCB (number of equivalents of added guest is shown at the right-hand end of each spectrum). Some regions of the spectrum where the changes are particularly clear are highlighted in green (diminishing peaks for free **H**^**w**^) and orange (emerging peaks for **H**^**w**^/1,2-DCB complex).

An example of a ^1^H NMR titration experiment involving addition of portions of 1,2-DCB to a solution of **H**^**w**^ is shown in Fig. 5. The paramagnetism of the high-spin Co(ii) ion disperses the signals over the range ±100 ppm,^11–14^ making it easy to see spectroscopic changes associated with a guest that is binding in slow exchange: the steady replacement of signals associated with free **H**^**w**^ (highlighted in green) by shifted signals associated with the formation of the **H**^**w**^/1,2-DCB complex during the titration (highlighted in orange) is clear. The value of *K* was determined by integration of these separate signals for free and complexed host, and knowledge of the concentration of all species present at each point.

We note that accurate deconvolution and integration of broad, overlapping signals from a paramagnetic complex can be difficult, so multiple individual integration measurements at different points in the spectra were averaged to reduce the experimental error. Moreover, the standard deviation from this averaging of multiple integration ratios has been doubled to provide an appropriately cautious estimate of experimental uncertainty, *i.e.* the error values in Table 1 are ±2σ.‡‡One of the reviewers pointed out that a common source of error in determination of binding constants from simple spectroscopic titrations is the simplistic use of concentrations rather than activities to quantify the species present, and in particular the fact that the activity of a fixed concentration of a species (host) can actually vary during a titration as more guest is added, to an extent depending on the solvent and the nature of the host and guest involved. Piguet and co-workers have looked at this in detail, see ref. 28. Whilst this will be as true in this paper as it is for the multitude of other cases where binding constants are calculated based on use of concentrations, it is the difficulty in deconvoluting and integrating broadened and overlapping signals in slow-exchange paramagnetic ^1^H NMR spectra that is the main source of error in this work. This is shown by the fact that we see significantly smaller errors associated with *K* values when they are derived from *e.g.* fluorescence measurements, or NMR measurements when the guest is in fast exchange: in such cases a large number of data points can be included in a conventional curve which is fit to a 1 : 1 isotherm (see *e.g.* ref. 13). The high (cautious) errors ascribed to the *K* values in Table 1, particularly in water where the guests were virtually insoluble and required 2% dmso to be present, do not however obscure the clear variations in *K* values with dipole moment, or the data extracted from the temperature-dependent van't Hoff plot, which are the main points of the paper. ^28^

**Table 1:** Summary of guest properties and binding constant data

Guest	Area/Å^2^	Volume/Å^3^	**H** ^**w**^ in D_2_O/DMSO-d^6^ (98 : 2, *v*/*v*)		**H** ^**PEG**^ in CD_2_Cl_2_	
			*K*/M^−1^	Δ*G*/kJ mol^−1^	*K*/M^−1^	Δ*G*/kJ mol^−1^
1,2-DCB	155.5	138.1	7000 ± 3000	−21.8 ± 1.2	45 ± 8	−9.4 ± 0.4
1,3-DCB	157.1	138.4	2700 ± 1400	−19.5 ± 1.3	9 ± 3	−5.3 ± 0.7
1,4-DCB	157.1	138.5	620 ± 340	−15.8 ± 1.4	a	a

a Binding too weak to measure by NMR spectroscopy.

From similar experiments with all three guests we obtained *K* values of 7000(±3000), 2700(±1400) and 620(±340) M^−1^ for the 1,2-, 1,3- and 1,4- isomers of DCB in water. The experimental uncertainties are high for the reasons given above, but (i) the values are in reasonable (order of magnitude) agreement with those predicted using GOLD, and (ii) the general trend is clear – and also as predicted by GOLD – with the binding constant decreasing in line with the reduced dipole moment across the series of guests. A plausible interpretation is that the δ- regions of the guests are those that lie closest to the positively charged cage surface, as the crystal structures all show, affording favourable electrostatic interactions. We know from other work with surface binding of anions that the high positive charge of the cage surface results in strong anion binding,^29^ which is the basis for the catalytic effects that we have seen.^4^ A similar favourable electrostatic interaction of the cage surface with a δ- part of a molecular dipole is quite possible here. Note that, although crystal structures show that two guests (for 1,2- and 1,3-DCB) can occupy a cage cavity under forcing and non-equilibrium conditions, we assume that binding of a second guest will be much weaker than the first, such that at the concentrations used the assumption that the speciation will be dominated by 1 : 1 host : guest complex formation is reasonable.^23^

**Fig. 6:**
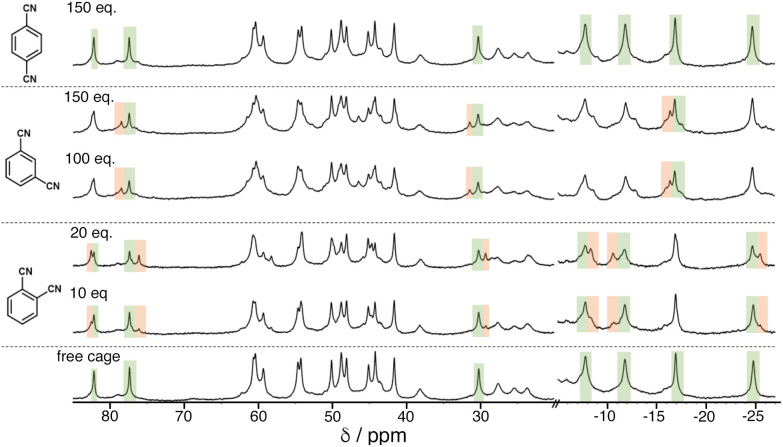
^1^H NMR titration experiment showing evolution of the NMR spectra of **H**^**PEG**^ (0.75 mM, 298 K) in CD_2_Cl_2_ on addition of increasing amounts of DCB isomers (number of equivalents of each added guest is shown on each spectrum). Some regions of the spectrum where the changes are particularly clear are highlighted in green (diminishing peaks for free **H**^**PEG**^) and orange (emerging peaks for **H**^**PEG**^/guest complexes).

We see the same effect of guest polarity, but with the background hydrophobic effect removed, by performing binding constant measurements for the three isomeric guests in CD_2_Cl_2_ rather than water. This necessitates use of the cage **H**^**PEG**^ with the same octanuclear core structure and cavity as **H** and **H**^**w**^ but bearing more solubilising substituents.^20^ It was immediately apparent that a far larger excess of the DCB guests was required to be able to observe new signals for the cage/guest complex (Fig. 6). Again deconvolution/integration of closely overlapping signals in the paramagnetic NMR spectra was non-trivial, an issue made worse because of the broader signals observed for **H**^**PEG**^ – a consequence of the 24 external chains and slower tumbling in solution. As before, signal pairs (for free and bound cage) were deconvoluted and integrated, and the resulting calculations of *K* were averaged over multiple measurements in different parts of the spectra and at different points during the titration. The resulting *K* values for 1 : 1 host/guest complex formation with the 1,2- and 1,3-DCB isomers were 45(±8) and 9(±3) M^−1^ respectively: with 1,4-DCB we observed no significant change in the NMR spectrum of **H**^**PEG**^ even after addition of >100 equivalents of 1,4-DCB (Fig. 6, top), meaning that binding of this guest in CD_2_Cl_2_ is too weak to measure by NMR spectroscopy. The same pattern as observed in water is clear, with guest binding correlating with polarity (1,2- > 1,3- > 1,4-DCB). Given the much less significant solvophobic contributions to guest binding in CH_2_Cl_2_ compared to water (see below), the effect of guest polarity dominates the binding constants more obviously.

From the binding constants in different solvents we can extract two interesting pieces of data associated with the effects of guest polarity. We assume that the similarity between the guests in other respects means that the dipole differences are the major factor in determining the binding constant differences – the assumption that underpinned the choice of guests. Firstly we note that, for the three measurements in water, the contribution to guest binding arising from polarity is approximately linear with dipole moment. The binding free energy of 1,4-DCB in water/2% dmso (−Δ*G* = 16 kJ mol^−1^) increases to 20 and then 22 kJ mol^−1^ for the 1,3- and 1,2-isomers respectively as the dipole moment increases from 0 to 4.3 to 7.1 Debye, *i.e.* an increase in −Δ*G* of 0.8 kJ mol^−1^ per Debye in this solvent. The second observation is that the effect of guest polarity is more pronounced in CD_2_Cl_2_ than in the aqueous solvent, with comparison between 1,3- and 1,2-DCB binding free energies showing an increment of 1.5 kJ mol^−1^ per Debye, nearly double the coefficient obtained in water/2% dmso. This likely reflects the fact that the dipoles of 1,2-DCB and 1,3-DCB are better stabilised by water than by CD_2_Cl_2_, meaning that binding of the more polar guests inside the cage cavity will carry a higher desolvation penalty in water/2% dmso than in CD_2_Cl_2_.

It is also interesting that 1,4-DCB shows no detectable binding to **H**^**PEG**^ in CD_2_Cl_2_, which implies that any favourable interactions between the guest and the cage interior surface (CH⋯π, van der Waals’, and CH⋯N hydrogen-bonding interactions) must be cancelled out by any desolvation costs and the free energy costs associated with combining two species into one supramolecular complex which restricts relative molecular motions.^8*b*,30^

In earlier work to quantify different contributions to binding with a range of guests we noted a significant contribution, in MeCN as solvent, from H-bonding between the guest and the cage interior surface in some cases.^11^ This is clearly not the case here, and we note that nitrile groups are significantly poorer hydrogen-bond acceptors, with a lower β parameter, than the functional groups such as amides and *N*-oxides that allowed H-bonding to be a significant contributor to guest binding in those earlier cases.^30,31^ Given this absence of significant binding of 1,4-DCB in CD_2_Cl_2_, and the relative lack of solvophobic effects in CD_2_Cl_2_ compared to water, one could reasonably conclude that the binding that we observe with more polar 1,3- and 1,2-DCB isomers in CD_2_Cl_2_ can be attributed to the additional polar contribution of a δ– region of the guest surface interacting with the 16+ cage surface.

In addition, given that there is no detectable binding of 1,4-DCB inside **H**^**PEG**^ in CD_2_Cl_2_ due to cancellation of the various favourable and unfavourable effects as described above, it follows that the binding free energy in water (−Δ*G* = 16 kJ mol^−1^) is ascribable solely to the change in solvent, *i.e.* a combination of the hydrophobic effect and any additional desolvation costs that apply in water. In previous work in which guests containing the same functional groups but zero or one additional fused aromatic rings were compared for their binding, we observed a consistent increment associated with binding of an additional aromatic ring in water compared to MeCN of *ca.* 10 kJ mol^−1^ due to the additional hydrophobic surface.^12^ The hydrophobic effect scales with surface area: 1,4-DCB (surface area 157 Å^2^) is significantly larger than one aromatic ring, and also is not purely hydrocarbon, but we just note here that the binding free energy of −Δ*G* = 16 kJ mol^−1^ for 1,4-DCB is approximately consistent with expectations based on previous work on the expected magnitude of hydrophobic contributions to binding of aromatic units.^12^

Overall the binding constant measurements in two different solvents clearly illustrate (i) the weakness of binding of non-polar 1,4-DCB in CD_2_Cl_2_ which means that binding is undetectable by NMR spectroscopy, (ii) the strength of the hydrophobic effect which drives binding in water, and (iii) the incremental consequence of guest polarity on binding free energy which is present in both solvents but is more pronounced in CD_2_Cl_2_.

### van't Hoff plots: dissection of guest binding contributions in water/2% dmso

To probe further the contributions to guest binding in water we performed variable-temperature NMR measurements on cage/guest mixtures which allowed us to see, through steady changes in the relative values of integrals associated with free and guest-containing cages, how the values of *K* changed with temperature. Standard van't Hoff plots of ln *K vs.* 1/*T* then allow the Δ*H* and Δ*S* contributions to guest binding to be separated. Given the magnitude of the binding constants this experiment only gives reasonable data in the water/2% dmso solvent system.

**Fig. 7:**
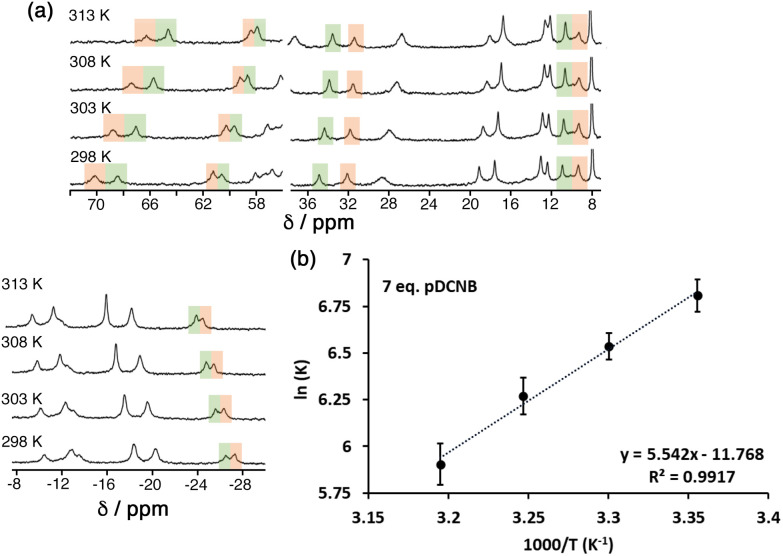
(a) Temperature-dependence of the NMR spectrum of a mixture of **H**^**PEG**^ and 1,4-DCB (7 equiv.) in D_2_O/DMSO-d^6^ showing the change in relative intensities between free **H**^**PEG**^ and the cage/guest complex as *K* changes with temperature. (b) van't Hoff plot based on this data, allowing determination of Δ*H* and *T*Δ*S* for guest binding (Table 2).

The results are summarised in Table 2 (see also Fig. 7), and it is immediately apparent that relatively small changes in −Δ*G* between the three guests are masking more substantial changes in Δ*H* and *T*Δ*S* which tend to oppose each other: this illustrates the phenomenon of ‘enthalpy/entropy compensation’^32^ whereby (in simple terms) a favourable change in enthalpy associated with a strong intermolecular interaction forming is offset by a loss of entropy associated with two independent species joining together. Here, the opposing changes do not quite cancel out. As the guest increases in polarity from 1,4-DCB to 1,2-DCB we see the modest steady increase in −Δ*G* that has been discussed earlier arises because of positive shifts in both Δ*H* (unfavourable) and *T*Δ*S* (favourable) that do not cancel, with the favourable increase in TΔ*S* more than compensating for the unfavourable Δ*H* change, so we can say that the increased guest binding associated with guest polarity is actually entropy-driven.

**Table 2:** Summary of thermodynamic data from the temperature-dependent NMR measurements, with the ΔΔ parameters reporting the effects of changes in guest polarity highlighted in bold

Guest	Δ*G*/kJ mol^−1^	ΔΔ*G*/kJ mol^−1^	Δ*H*/kJ mol^−1^	ΔΔ*H*/kJ mol^−1^	*T*Δ*S* /kJ mol^−1^	Δ(*T*Δ*S*)/kJ mol^−1^
1,2-DCB	−21.8		−30.5		−8.7	
		**+2.9**		**−12.6**		**−15.4**
1,3-DCB	−18.9^a^		−43.1		−24.1	
		**+2.0**		**−3.0**		−5.1
1,4-DCB	−16.9^a^		−46.1		−29.2	

a These Δ*G* values are slightly different from those in column 5 of Table 1 (though not significantly) as they were recorded in separate experiments as part of the temperature-dependent series. In particular only one host : guest ratio was used for the van't Hoff experiments, whereas the Δ*G* values in Table 1 are based on a larger number of signal integrations at a range of different host : guest ratios.

Based on the preceding discussion, this direction for the Δ*H* and *T*Δ*S* changes on guest binding in water is counter-intuitive: the polarity effect that we proposed earlier, *viz.* that an increased dipole on the guest provides the opportunity for δ– regions of the guest to interact favourably with the cationic cage surface, would constitute a favourable Δ*H* contribution to guest binding. Whilst this remains a likely contribution to the binding of the more polar guests, it appears to be small (*cf.* the small binding constants in CH_2_Cl_2_) and masked by larger and less predictable changes in the Δ*H* and Δ*S* contributions to the hydrophobic effect associated with structural changes in the guests. Specifically the polar guest 1,2-DCB is expected to be more strongly solvated in water than non-polar 1,4-DCB, resulting in a greater enthalpy penalty for desolvation compared to 1,4-DCB: conversely, liberation of the tighter-bound solvation sphere from around 1,2-DCB will result in a larger entropy gain than occurs from more weakly-solvated 1,4-DCB. This is enthalpy/entropy compensation again,^32^ but the opposite way around to the simple example described earlier, and the entropy effect wins in controlling changes in binding free energies across this series of guests in water.

Importantly we can expect that the entropy decrease associated with a guest binding inside the cage cavity will be similar with each guest;^8*b*,30^ and the number of water molecules liberated from the cavity following guest binding will be similar in each case given that the molar volumes of the three isomeric guests are so similar. This leaves desolvation of the guests on binding as the main variable to account for the trend in *K* values that we observed.

We note that whilst the hydrophobic effect was originally considered as primarily entropic in origin,^33^ much recent work has shown that it can have a substantial enthalpy contribution,^14,34^ with the balance between the two effects being unpredictable. We observed a while ago that the improved binding free energy of guests inside **H**^**w**^ associated with addition of a hydrophobic CH_2_ group to the guest skeleton was mostly *enthalpic* in origin.^14^ We also note that the ΔΔ*H* and Δ(*T*Δ*S*) values associated with the change from 1,3-DCB to 1,2-DCB are much larger than those associated with the change from 1,4-DCB to 1,3-DCB despite the slightly smaller dipole moment increment, for which there is no simple explanation.

Overall, in this guest series, it is clear that the hydrophobic effect is the dominant thermodynamic contribution to guest binding in water, as shown by differences in −Δ*G* between CD_2_Cl_2_ and water. The different Δ*H*/*T*Δ*S* contributions to guest binding across the guest series in water arise principally from changes in solvation of the guest when it binds, and cannot be rationalised simply by considering direct cage/guest electrostatic interactions. We note that Raymond and co-workers came to similar conclusions regarding the dominance of guest desolvation on controlling binding affinities for a wide range of guests inside a coordination cage host in protic solvents.^10*a,b*^

## Conclusions

This set of isomeric guests provides changes in dipole moment whilst keeping as fixed as possible molecular volume, surface area, and the functional groups involved in hydrogen-bonding, allowing us to evaluate the effects of guest polarity on binding inside a coordination cage host in water/2% dmso, and CD_2_Cl_2_. Overall it is clear that increases in guest dipole moment results in increased binding free energies −Δ*G*, but the magnitudes of any polarity-induced changes in binding strength are small compared to the hydrophobic effect which dominates guest binding in water. In water, in terms of Δ*H* and *T*Δ*S* components, the increased free energy of guest binding for the more polar guests is entropy-based, with the enthalpy changes associated with binding of the more polar guests being unfavourable but less significant. We ascribe this to the effects of guest desolvation, with the more polar guests that have a tighter solvation sphere in water needing a higher enthalpy penalty to desolvate them, but also showing a greater favourable entropy change when those solvent molecules are liberated to allow guest binding to occur. In water/2% dmso, any favourable direct interactions between polar guests and the cage (local δ+/δ– interactions that become stronger as guest dipole moment increases) are therefore less significant in determining the pattern of guest binding free energies across the series than is guest desolvation which is the dominant effect.

## Experimental details

Samples of **H** (used for crystalline sponge experiments),^19^**H**^**w**^ (used for guest binding studies in water/2% dmso)^12^ and **H**^**PEG**^ (used for guest binding studies in CD_2_Cl_2_)^20^ were prepared according to published methods. The three isomers of DCB were purchased from Sigma-Aldrich and used as received. Calculated binding constants for the DCB isomers (assuming pure water as solvent) using the GOLD programme were performed as outlined in ref. 16. Details of the methodology used for the NMR titrations to determine guest binding constants, and the temperature-dependent measurements in water/2% dmso, are in the ESI.† Molecular surface areas and volumes for the three guests were calculated using the iSpartan app from Wavefunction, Inc.

The crystalline sponge experiments were performed as described in a previous paper,^23^ by immersing pre-grown crystals of **H** (as the tetrafluoroborate salt)^19^ into a concentrated MeOH solution of the relevant guest. Information on the crystal properties, data collections and refinements associated with the structure determinations of the cage/guest complexes of **H** are collected in Table S1 of ESI.† The data collections were performed in Experiment Hutch 1 of beamline I–19 at the UK Diamond Light Source synchrotron facility,^35^ using methodology, data processing and software described previously.^23^

## Author contributions

C. M.: Synthesis and NMR measurements of cage/guest binding constants. A. B. S. and C. G. P. T.: crystalline sponge experiments and X-ray crystallography. B. S.: calculations of predicted binding constants using GOLD. M. D. W.: project conception and supervision, manuscript preparation.

## Conflicts of interest

There are no conflicts to declare.

## Supplementary Material

DT-051-D2DT02623F-s001

DT-051-D2DT02623F-s002
