# Correction: Disentangling contributions to guest binding inside a coordination cage host: analysis of a set of isomeric guests with differing polarities

**DOI:** 10.1039/d3dt90102e

**Published:** 2023-06-14

**Authors:** Cristina Mozaceanu, Atena B. Solea, Christopher G. P. Taylor, Burin Sudittapong, Lidón Pruñonosa Lara, Michael D. Ward

**Affiliations:** a Department of Chemistry, University of Warwick Coventry CV4 7AL UK m.d.ward@warwick.ac.uk

## Abstract

Correction for ‘Disentangling contributions to guest binding inside a coordination cage host: analysis of a set of isomeric guests with differing polarities’ by Cristina Mozaceanu *et al.*, *Dalton Trans.*, 2022, **51**, 15263–15272, https://doi.org/10.1039/D2DT02623F.

The authors regret the omission of **Lidón Pruñonosa Lara**, who contributed significantly to the crystallography and should have been included as a co-author.

The corrected list of authors and affiliations for this paper is as shown above.

The Royal Society of Chemistry apologises for these errors and any consequent inconvenience to authors and readers.

## Supplementary Material

