# Supplementary material for: Disentangling contributions to guest binding inside a coordination cage host: analysis of a set of isomeric guests with differing polarities
Source: Dalton Trans. 2022 Sep 16;51(40):15263–72. doi: 10.1039/d2dt02623f (PMC9578013; doi:10.1039/d2dt02623f)
Supplement: DT-051-D2DT02623F-s001 [file DT-051-D2DT02623F-s001.pdf]

Electronic Supplementary Material (ESI) for Dalton Transactions.
This journal is © The Royal Society of Chemistry 2022

Disentangling contributions to guest binding inside a coordination cage host: analysis of a set of isomeric guests with differing polarities

Cristina Mozaceanu, Atena B. Solea, Christopher G. P. Taylor, Burin Sudittapong and Michael D. Ward

Supporting information

1. Procedure for variable temperature ^{1}H paramagnetic NMR experiments

The variable temperature ^{1}H paramagnetic NMR experiments were carried out on a Bruker Avance III 400 MHz instrument, at NS 1024. For 1,2-DCB and 1,3-DCB, spectra were recorded in the range of 298 – 358 K with a 10 K temperature step, whilst for 1,4-DCB in the range of 298 – 328 K with a 5 K step, respectively.

A 0.20 – 0.25 mM stock of host (**H^W**) was prepared in D_2O by stirring and heating the solution for 2 h at 80 °C. The concentration of the resulting post-filtration solution was determined using the Lambert-Beer law, knowing the extinction coefficient of **H^W** in water, $2.77 \cdot 10^5$ M^{-1} cm^{-1}.

The guest stock solutions prepared in 100% DMSO-d^6 and the mixed solutions in tubes – 500 µL final volume, DMSO-d^6:D_2O 2:98 v:v – were prepared according to Table S1. Guest equivalents in the stock solutions were represented by the $[G]_s/[H]_0$ ratio, where $[H]_0$ is the concentration of **H^W** in the tube, and $[G]_s$ is the concentration of the guest stock solution. The final guest solutions in the tubes were 50 times diluted compared to the guest stocks. The species distribution in each tube at equilibrium was approx. 50:50 HG:H.

Table S1. Sample preparation for the variable temperature experiments.

Guest	1,2-DCB	1,3-DCB	1,4-DCB
**Equivalents of guest in the guest stock solution**	50	100	350
**Equivalents of guest in the tubes**	1	2	7
**Added volume of guest stock solution (µL)**	10	10	10
**Added volume of host stock solution (µL)**	490	490	490

All solutions were added in tubes with Hamilton 700 series syringes, then they were heated at 75 °C for 1 h for equilibration. Each experiment was repeated two times and the signals were processed as follows.

For each spectrum, several peak pairs (Figures S1-S3; green boxes – free host, orange boxes – host-guest) were deconvoluted in the MestReNova software with a mixed Lorentzian/Gaussian model manually adjusted to ensure a good fit. The values of the deconvoluted areas were included in the calculation of the average binding constants (K_{avg}) at a specific temperature. The Gibbs free energy of binding was calculated for K_{avg} at 298 K. All statistics were carried out using the 'Descriptive Statistics' included in the 'Data Analysis' add-on in Excel. Van't Hoff plots for each guest were depicted in Figures S4-S6. For each K_{avg} a positive ($error_+$) and a negative ($error_-$) error were determined using the equations below, where *s.d.* represents the standard deviation of the data set. The error bars represent ± the absolute value of the $error_-$ which was higher than $error_+$. Each Van't Hoff plot was fitted to a linear regression, which was further used to determine the thermodynamic parameters of the system collected in Table 2 (main text).

$$error_+ = \ln\left(K_{avg}\right) - \ln\left(K_{avg} + 2 \cdot s.d.\right)$$

$$error_- = \ln\left(K_{avg}\right) - \ln\left(K_{avg} - 2 \cdot s.d.\right)$$

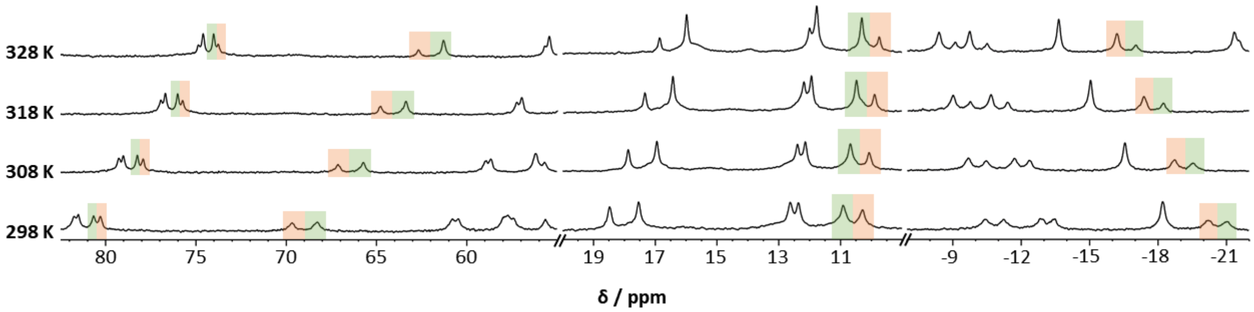

Figure S1. Partial VT NMR spectra of 1 eq. 1,2-DCB/$\mathbf{H_o}$ in 2% DMSO-d^6/D_2O, where green boxes – free host, orange boxes – host-guest.

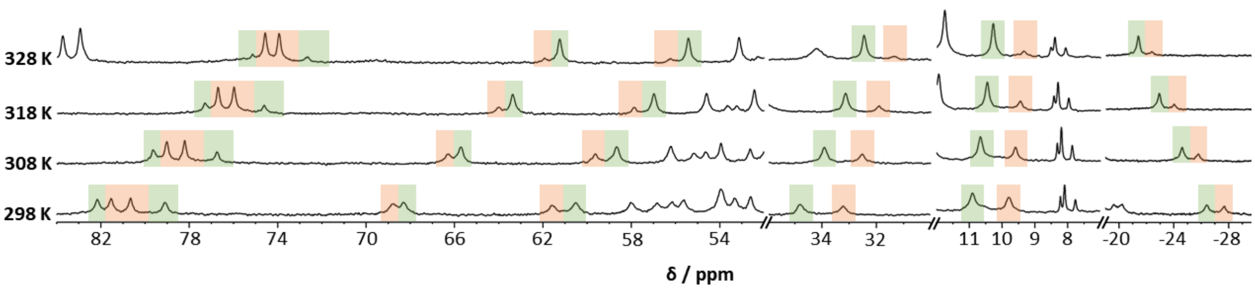

Figure S2. Partial VT NMR spectra of 2 eq. 1,3-DCB/$\mathbf{H^W}$ in 2% DMSO-d^6/D_2O, where green boxes – free host, orange boxes – host-guest.

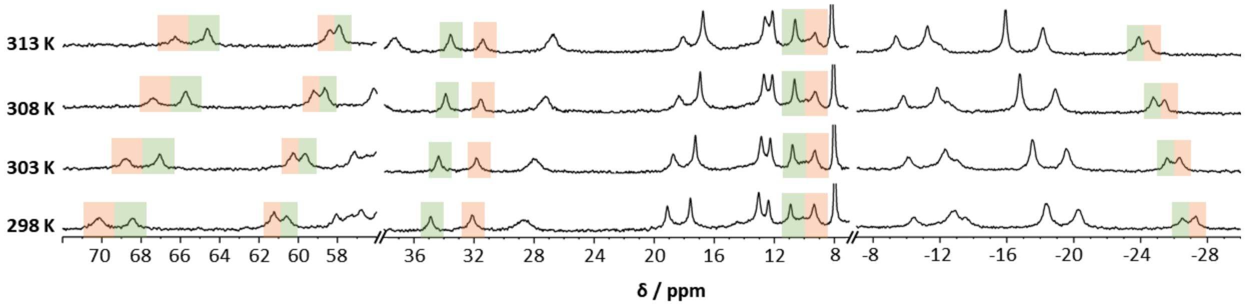

Figure S3. Partial VT NMR spectra of 7 eq. 1,4-DCB/$\mathbf{H^W}$ in 2% DMSO-d^6/D_2O, where green boxes – free host, orange boxes – host-guest.

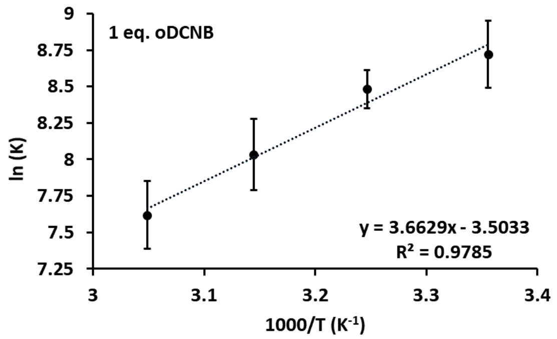

Figure S4. Van't Hoff plot corresponding to 1 eq. 1,2-DCB/$\mathbf{H^W}$ in 2% DMSO-d^6/D_2O at 298 – 328 K.

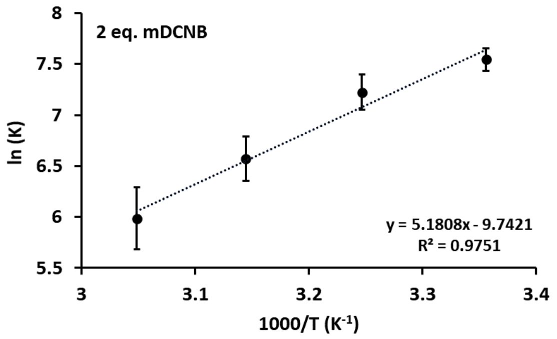

Figure S5. Van't Hoff plot corresponding to 2 eq. 1,3-DCB/$\mathbf{H^W}$ in 2% DMSO-d^6/D_2O at 298 – 328 K.

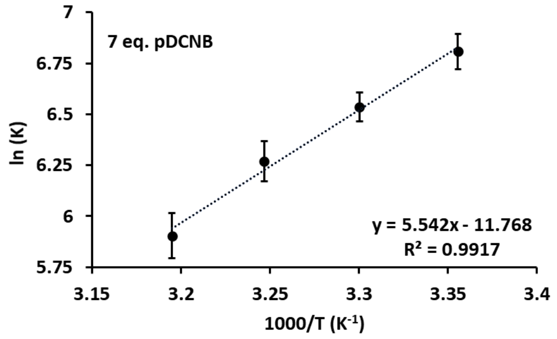

Figure S6. Van't Hoff plot corresponding to 7 eq. 1,4-DCB/$\mathbf{H^W}$ in 2% DMSO-d^6/D_2O at 298 – 313 K.

2. Procedure for ^{1}H paramagnetic NMR experiments in 2% DMSO-d^6/D_2O, 298 K

The ^{1}H paramagnetic NMR experiments were carried out on a Bruker Avance III 300 MHz instrument for 1,2-DCB and 1,3-DCB, and on a Bruker Avance III 400 MHz instrument for 1,4-DCB at NS 1024, 298 K.

All solutions and tubes were prepared in a similar manner as described above. A 0.20 – 0.25 mM stock of host (**H^W**) was prepared in D_2O. The stock guest solutions were prepared in 100% DMSO-d^6. *Table S2* illustrates the concentrations and volumes used in the sample preparation for these experiments. Guest equivalents in the stock solutions were represented by the $[G]_s/[H]_0$ ratio, where $[H]_0$ is the concentration of **H^W** in the tube, and $[G]_s$ is the concentration of the guest stock solution. Each tube contained 500 μL final volume, DMSO-d^6:D_2O 2:98 v:v. In each tube, the stock of guest was diluted with a solution of DMSO-d^6 up to a total volume of 10 μL to achieve the right equivalents of guest. The added volume of host stock in the tube was 490 μL. This way, in the tube there was only 2% DMSO-d^6.

Table S2. Sample preparation for the NMR experiments in 2% DMSO-d^6/D_2O, 298 K.

Guest	1,2-DCB	1,3-DCB	1,4-DCB
**Equivalents of guest in the guest stock solution**	125	250	500
**Equivalents of guest in the tubes**	0 – 2.5	0 – 5	0 – 10
**Added volume of guest stock solution (μL)**	0 – 10	0 – 10	0 – 10
**Added volume of DMSO-d^6 (μL)**	10 – 0	10 – 0	10 – 0
**Added volume of host stock solution (μL)**	490	490	490

The signal processing was carried out in a similar manner as described above. Each experiment was repeated two times. The values of the average binding affinities and Gibbs free energies are collected in the ain text (Table 1) reported with an uncertainty of two standard deviations.

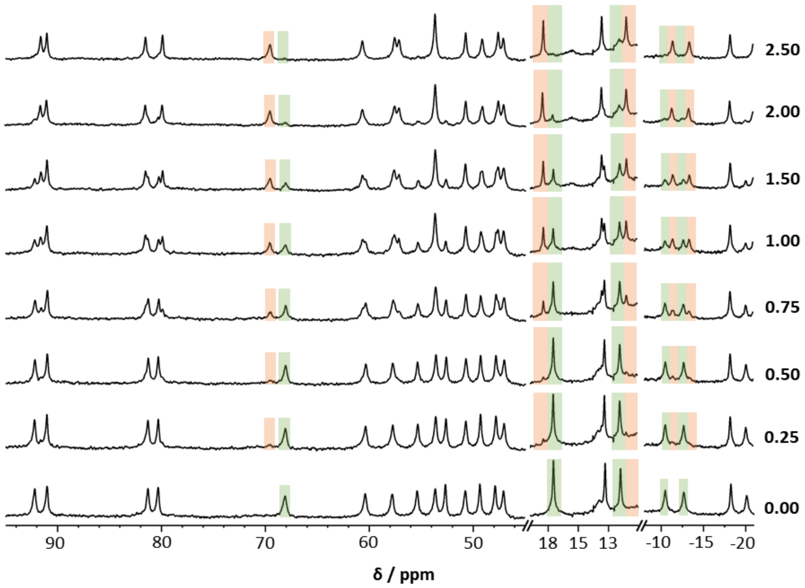

Figure S7. Partial NMR spectra of 1,2-DCB/**H^W** in 2% DMSO-d^6/D_2O (0–2.5 eq., bold text, right; 298 K), where green boxes – free host, orange boxes – host-guest.

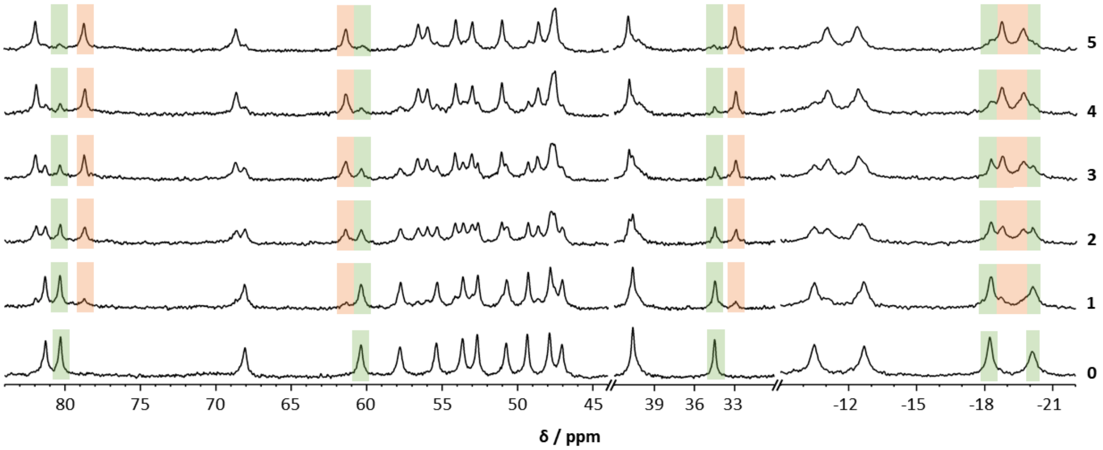

Figure S8. Partial NMR spectra of 1,3-DCB/$\mathbf{H^{W}}$ in 2% DMSO-d^6/D_2O (0–5 eq., bold text, right; 298 K), where green boxes – free host, orange boxes – host-guest.

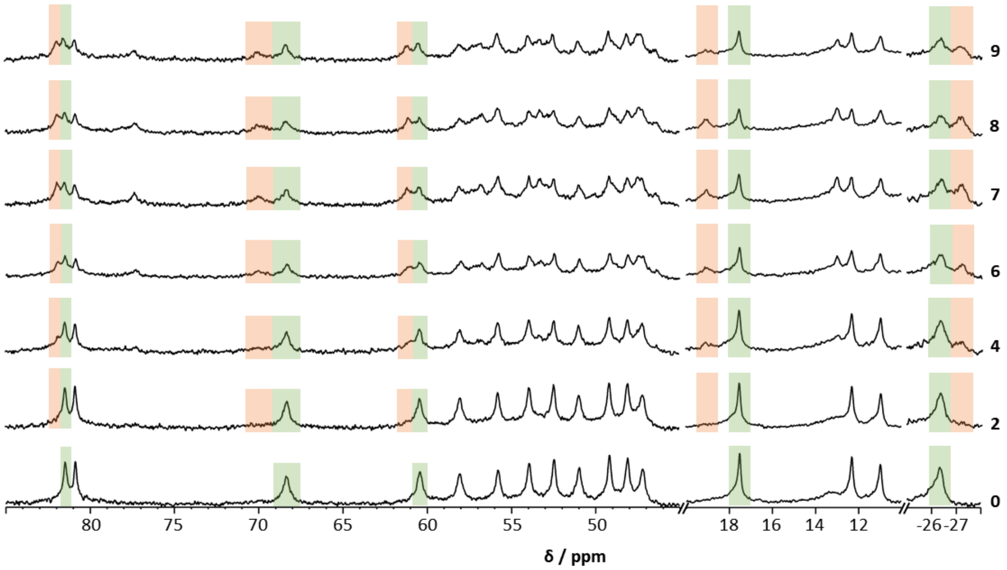

Figure S9. Partial NMR spectra of 1,4-DCB/$\mathbf{H^{W}}$ in 2% DMSO-d^6/D_2O (0–9 eq., bold text, right; 298 K), where green boxes – free host, orange boxes – host-guest.

3. Procedure for ^{1}H paramagnetic NMR experiments in CD_2Cl_2, 298 K

The ^{1}H paramagnetic NMR experiments were carried out on a Bruker Avance III 400 MHz instrument at NS 2048, 298 K.

A fresh 0.7 – 0.8 mM stock host ($\mathbf{H^{PEG}}$) solution in CD_2Cl_2 was prepared for each experiment. The concentration of the stock host solution was determined through the Lambert-Beer law using a pre-determined extinction coefficient of $\mathbf{H^{PEG}}$ in 1,2-dichloroethane, $2.87{\cdot}10^5$ M^{-1} cm^{-1}.

Stock solutions of guests were prepared at different equivalents directly in the stock host, by dissolving accurate amounts of guests: i) 1,2-DCB 20 equivs., ii) 1,3-DCB 150 equivs., and iii) 1,4-DCB 150 equivs. These

were allowed to equilibrate for 10 min at 4 °C to prevent evaporation. For 1,2-DCB and 1,3-DCB, a second tube was prepared by diluting the stock of guest with the right volume of stock host to reach 10 equivs. 1,2-DCB, and 100 equivs. 1,3-DCB, respectively. No second tube was prepared for 1,4-DCB, as no guest binding was observed and higher equivalents could not be reached due to solubility limits. All solutions were added in tubes with Hamilton 700 series syringes.

Each experiment was repeated two times and the signals were processed in a similar manner as described above. The values of the average binding affinities and Gibbs free energies are collected in the main text (Table 1), reported with an uncertainty of two standard deviations.

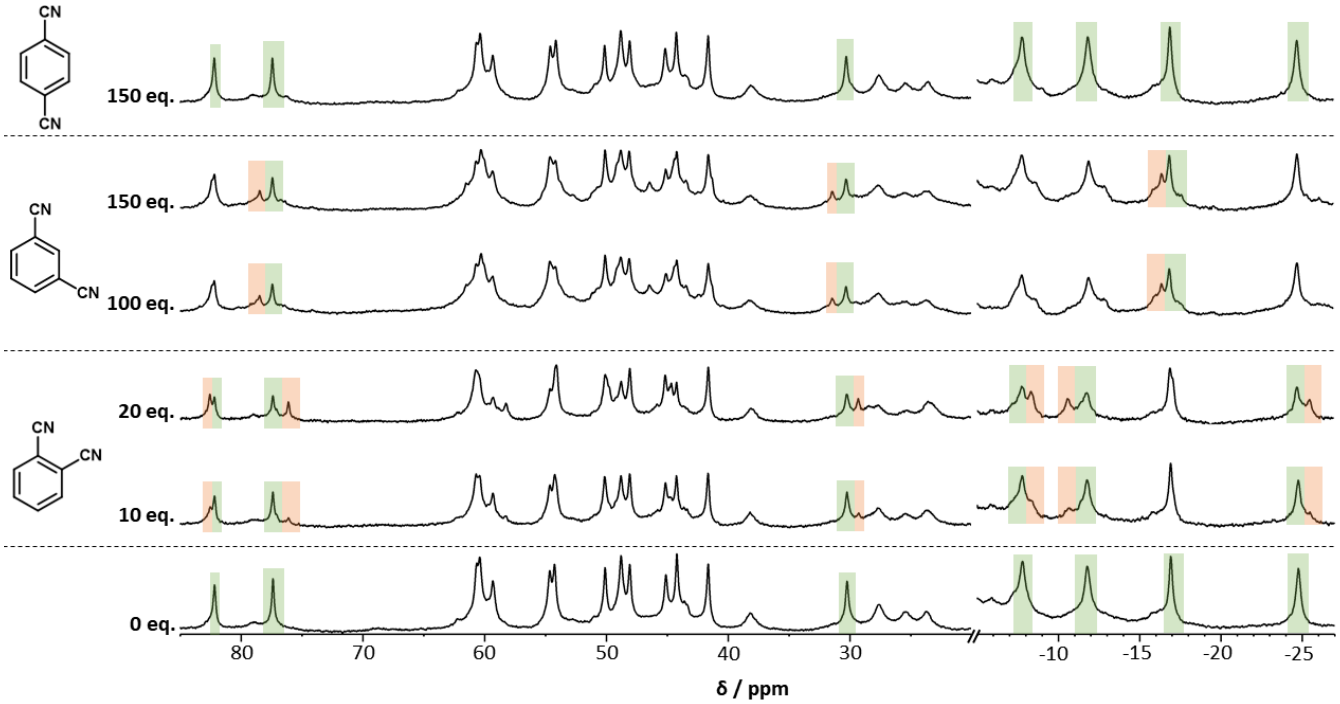

Figure S10. Partial NMR spectra of host-guest complexes at different guest equivalents (bold text, left), 298 K. Green boxes – free host, orange boxes – host-guest.

4. Crystallographic details

Details of the crystals used, data collection and refinements are given in Table S4 (below). As is usual with crystallographic structure determinations of this kind on elaborate supramolecular assemblies, scattering is weak and refinement problems are significant due to substantial disorder, principally of anions and solvent molecules, but also – in two of these cases – of cavity-bound guests. The cage superstructure itself is generally well-behaved in all cases. These problems required (i) extensive use of restraints to ensure geometrically reasonable structures, and (ii) elimination of regions of diffuse electron density using the solvent mask feature in OLEX leaving apparent voids in the lattices. Details pertaining to each structure are included in the individual CIFs. Discussion of the structures in the main text is accordingly at the level of demonstrating the presence, position and site occupancy of particular guests in their cavity; detailed discussion of structural minutiae is kept to a minimum.

Table S3. Summary of crystal parameters, data collections and refinement details for the three crystal structures

Structure name	**H•12DCB**	**H•13DCB**	**H•14DCB**
CCDC number	2194795	2194796	2194797
Empirical formula	$B_{15.5}C_{385}Cl_{0.5}Co_8F_{62}H_{404}N_{76}O_{33}$	$B_{15}C_{380}ClCo_8F_{60}H_{414}N_{76}O_{43}$	$B_{16}C_{371}Co_8F_{64}H_{390}N_{73}O_{31}$
Formula weight	8458.54	8542.89	8227.94
T / K	100	100	100
Radiation	Synchrotron (λ = 0.6889 Å)	Synchrotron (λ = 0.6889 Å)	Synchrotron (λ = 0.6889 Å)
Crystal system	monoclinic	monoclinic	monoclinic
Space group	*C*2/c	*C*2/c	*C*2/c
a/Å	32.92731(7)	32.85293(12)	32.9780(3)
b/Å	30.26746(6)	30.18243(10)	29.72814(19)
c/Å	39.97143(8)	40.21139(14)	39.5702(3)
β/°	96.20586(17)	95.6471(3)	96.1393(7)
Volume/$Å^3$	39603.12(10)	39679.35(17)	38571.1(4)
Z	4	4	4
ρ_{calc} / g cm^3	1.419	1.430	1.417
μ/mm^{-1}	0.402	0.406	0.408
Crystal size/mm^3	0.12 x 0.11 x 0.09	0.08 x 0.07 x 0.06	0.11 x 0.10 x 0.09
2Θ range for data collection/°	1.97 – 59.894	1.97 – 58.042	2.01 – 59.894
Reflections collected	338129	579320	331209
Independent reflections/R_{int}	62969 / 0.0456	58012 / 0.0436	61372 / 0.0621
Data/restraints/parameters	62969 / 9242 / 2538	58012 / 5731 / 2360	61372/8379/2481
Goodness-of-fit on F^2	1.095	1.103	1.040
Final R_1 / wR_2 ^{a}	0.0534, 0.1855	0.0593, 0.2103	0.0680, 0.2330
Largest diff. peak/hole / e $Å^{-3}$	1.13 / -0.58	2.00 / -0.82	1.91 / –0.68

[a] The value of *R*1 is based on 'observed' data with $I > 2\sigma(I)$; the value of w*R*2 is based on all data.
